# Tumour-specific HMG-CoAR is an independent predictor of recurrence free survival in epithelial ovarian cancer

**DOI:** 10.1186/1471-2407-10-125

**Published:** 2010-04-01

**Authors:** Donal J Brennan, Jenny Brändstedt, Elton Rexhepaj, Michael Foley, Fredrik Pontén, Mathias Uhlén, William M Gallagher, Darran P O'Connor, Colm O'Herlihy, Karin Jirstrom

**Affiliations:** 1 Dept of Obstetrics and Gynaecology, National Maternity Hospital, Holles Street, Dublin 2, Ireland; 2 UCD School of Biomolecular and Biomedical Science, UCD Conway Institute, University College Dublin, Dublin, Ireland; 3 Center for Molecular Pathology, Department of Laboratory Medicine, Malmö University Hospital, Lund University, Malmö, Sweden; 4 UCD School of Medicine and Medical Science, National Maternity Hospital, Holles Street, Dublin 2, Ireland; 5 Department of Genetics and Pathology, Rudbeck Laboratory, Uppsala University, Uppsala, Sweden; 6 Department of Biotechnology, AlbaNova University Center, Royal Institute of Technology, Stockholm, Sweden; 7 CREATE Health Center for Translational Cancer Research, Lund University, Lund, Sweden

## Abstract

**Background:**

Our group previously reported that tumour-specific expression of the rate-limiting enzyme in the mevalonate pathway, 3-hydroxy-3-methylglutharyl-coenzyme A reductase (HMG-CoAR) is associated with more favourable tumour parameters and a good prognosis in breast cancer. In the present study, the prognostic value of HMG-CoAR expression was examined in tumours from a cohort of patients with primary epithelial ovarian cancer.

**Methods:**

HMG-CoAR expression was assessed using immunohistochemistry (IHC) on tissue microarrays (TMA) consisting of 76 ovarian cancer cases, analysed using automated algorithms to develop a quantitative scoring model. Kaplan Meier analysis and Cox proportional hazards modelling were used to estimate the risk of recurrence free survival (RFS).

**Results:**

Seventy-two tumours were suitable for analysis. Cytoplasmic HMG-CoAR expression was present in 65% (n = 46) of tumours. No relationship was seen between HMG-CoAR and age, histological subtype, grade, disease stage, estrogen receptor or Ki-67 status. Patients with tumours expressing HMG-CoAR had a significantly prolonged RFS (p = 0.012). Multivariate Cox regression analysis revealed that HMG-CoAR expression was an independent predictor of improved RFS (RR = 0.49, 95% CI (0.25-0.93); p = 0.03) when adjusted for established prognostic factors such as residual disease, tumour stage and grade.

**Conclusion:**

HMG-CoAR expression is an independent predictor of prolonged RFS in primary ovarian cancer. As HMG-CoAR inhibitors, also known as statins, have demonstrated anti-neoplastic effects *in vitro*, further studies are required to evaluate HMG-CoAR expression as a surrogate marker of response to statin treatment, especially in conjunction with current chemotherapeutic regimens.

## Background

Epithelial ovarian cancer (EOC) is the leading cause of death from gyneacological malignancy and the fifth most common cause of cancer-related death in women. In 2008 it was estimated that 21,650 new ovarian cancer cases will be diagnosed in the United States and that 15,520 will succumb to the disease [1]. Despite improvements in surgical techniques and the advent of more targeted therapeutics such as bevacizumab, survival of patients with EOC stands at 45% at five years [1]. Such poor statistics indicate an urgent requirement to improve our understanding of the molecular mechanisms underlying EOC, which may lead to the development of improved prognostic and predictive assays.

3-hydroxy-3methylglutharyl-coenzyme A reductase (HMG-CoAR) acts as a rate-limiting enzyme in the mevalonate pathway. Although cholesterol represents the main product of this pathway, it also produces a number of non-sterol isoprenoid side products, which have been shown to have a number of tumour-suppressive properties [2] and to be important regulators of angiogenesis, proliferation, and migration [3,4]. HMG-CoAR inhibitors (statins), have demonstrable anti-neoplastic effects *in vitro *[5-7] and in xenograft models [7]. Both the isoprenoid-mediated anti-tumoural properties, and the cholesterol-reducing effects of statins have been suggested to lower the cancer incidence among statin users [8], although, to date, epidemiological studies have been unable to confirm an association between statin therapy and ovarian cancer risk [9-11].

Members of our group have previously investigated tumour-specific expression of HMG-CoAR by immunohistochemistry (IHC) in 511 incident breast cancer cases within the population-based prospective cohort Malmö Diet and Cancer Study [12]. This study demonstrated that HMG-CoAR was expressed at various intensities in 82% of the tumours and increased levels of HMG-CoAR protein expression were associated with favourable characteristics, such as a smaller tumour size, low histological grade and estrogen receptor (ER) positivity [13]. A validation study confirmed these findings and demonstrated that HMG-CoAR was an independent prognostic marker, associated with an improved recurrence free survival (RFS) [14].

Based on these data, the prognostic power of tumour-specifc HMG-CoAR expression in EOC was examined. This study describes the use of tissue microarray (TMA) technology to investigate the prognostic value of HMG-CoAR in EOC and the use of automated image analysis to quantify HMG-CoAR expression.

## Methods

### Patients and tumour samples

Prior to commencing the study a power calculation revealed that a cohort of 54 patients would allow for a power of 0.95 (G*Power, http://www.psycho.uni-duesseldorf.de/aap/projects/gpower/). The TMA, used in this study was constructed from a consecutive cohort of 76 patients diagnosed with primary invasive epithelial ovarian cancer at the National Maternity Hospital, Dublin, with a median follow-up of 4.3 years. The patient cohort has been described previously [15]. The standard surgical management was a total abdominal hysterectomy, bilateral salpingo-oophorectomy and omentectomy with cytological evaluation of peritoneal fluid or washings. Residual disease was resected to less than 2 cm where possible. Stage and volume of residual disease (no residual disease, residual disease greater or less than 2 cm) were recorded in all cases. All patients received adjuvant chemotherapy consisting of cisplatin or carboplatin prior to 1992 and combined with paclitaxel from 1992 to 2002. No patient received neo-adjuvant chemotherapy. Benign or borderline ovarian cancers, non-epithelial ovarian cancer and cases with histological features typical of secondary ovarian cancer were excluded from the study. Diagnostic specimens were all formalin fixed and paraffin embedded in the Department of Pathology at the National Maternity Hospital, Dublin, Ireland. All tissue blocks were stored in that department prior to construction of the TMA. Full ethical approval was obtained from the Ethics Committee of the National Maternity Hospital, Dublin and informed consent was obtained from living patients and relatives of deceased patients.

### Tissue microarrays and immunohistochemistry

Seventy six paraffin-embedded tumour specimens were used for tissue microarray (TMA) construction as previously described [15]. Areas representative of invasive cancer were marked on haematoxylin and eosin-stained slides and the TMA was constructed, using a manual tissue arrayer (MTA-1, Beecher Inc, WI). The array consisted of four cores per patient. Two 1.0 mm cores were extracted from each donor block and assembled in a recipient block. Recipient blocks were limited to approximately 100 cores each. In general, cores were taken from the peripheral part of the tumour in cases where the tumour had well-defined borders. In more diffusely growing tumours, areas with the highest tumour cell density were primarily targeted. Necrotic tissue was avoided.

Four μm sections were automatically pretreated using the PT-link system (DAKO, Copenhagen, Denmark) before being stained in a Techmate 500 (DAKO, Copenhagen, Denmark) with a polyclonal anti-HMG-CoAR antibody (Upstate, Lake Placid, NY) diluted 1:250 as described previously [14]. Cytoplasmic staining of HMG-CoAR was assessed according to intensity (negative - 0, weak - 1, moderate - 2, strong - 3). When present, HMG-CoAR was generally expressed in the majority of tumour cells (> 50%) and therefore, only the staining intensity was accounted for in the manual analyses.

### Image Acquisition, Management and Automated analysis

The Aperio ScanScope XT Slide Scanner (Aperio Technologies, Vista, CA) system was used to capture whole slide digital images with a 20× objective. Slides were de-arrayed to visualize individual cores, using Spectrum (Aperio). Genie™ histology pattern recognition software (Aperio) was used to identify tumour from stroma in individual cores and a colour deconvolution algorithm (Aperio) was used to quantify tumour-specific HMG-CoAR expression. Estrogen receptor and Ki-67 were analyzed using a previously described algorithm [16] and a 10% threshold was used for dichomotization of data.

### Statistical analysis

Spearman's Rho correlation was used estimate the relationship between cores from individual tumours, Pearson correlation was used to estimate the relationship between manual and automated analysis. Differences in distribution of clinical data and tumour characteristics between samples with a high and low HMG-CoAR expression (described below) were evaluated using the χ^2 ^test. Kaplan-Meier analysis and the log rank test were used to illustrate differences between RFS and overall survival (OS). Cox regression proportional hazards models were used to estimate the relationship between survival and HMG-CoAR, residual disease, stage and grade. All calculations were performed, using SPSS version 11.0 (SPSS Inc, Chicago, IL). P values < 0.05 were considered statistically significant.

## Results

### Immunohistochemical Expression of HMG-CoAR in Epithelial Ovarian Cancer

**Figure 1:**
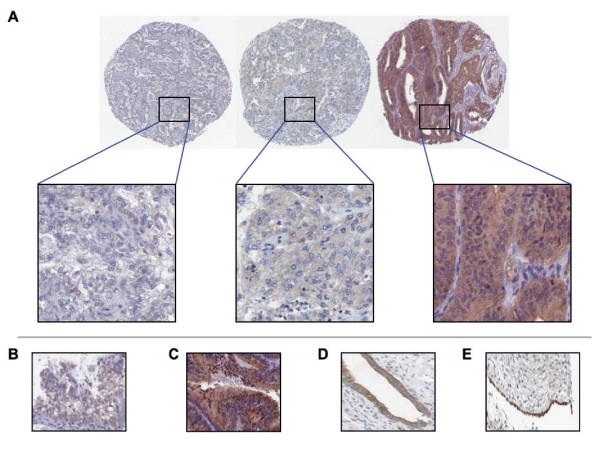
**HMG-CoAR Expression in EOC**. Examples of immunohistochemical HMG-CoAR staining in EOC with negative, intermediate and strong cytoplasmic expression (5× and 20× magnification) (A). Areas of membranous expression (B) and granular staining (C) were also seen (20× magnification). HMG-CoAR expression was also evident in normal fallopian tube (D) and normal ovarian surface epithelium (E) (20× magnification).

HMG-CoAR expression was evaluable in 72 of 76 cases (95%). The remaining cores were lost during antibody optimisation and staining. HMG-CoAR was generally confined to tumour epithelium and was expressed in various intensities in the cytoplasm (Fig. 1A). Stromal expression of HMG-CoAR was not seen. Only staining intensity was accounted for in statistical analysis of HMG-CoAR protein expression, as when present, HMG-CoAR was generally expressed in the majority of tumour cells (> 50%), a finding consistent with previous studies[13,14]. Nuclear expression of HMG-CoAR was not detected; however, membranous expression of HMG-CoAR was evident in a small number of cases (Fig. 1B). Granular cytoplasmic staining was also seen in a small number of cases (Fig. 1C) Twenty-five of the 72 tumours (35%) lacked HMG-CoAR expression, 35 (47%) demonstrated weak, 12 (18%), moderate and none demonstrated a strong signal. HMG-CoAR expression was also examined in a panel of normal ovarian and fallopian tube specimens. HMG-CoAR expression was seen in the majority of normal ovarian and fallopian tube epithelium (Fig. 1D and 1E).

### HMG-CoAR is Associated with an Improved Prognosis

**Figure 2:**
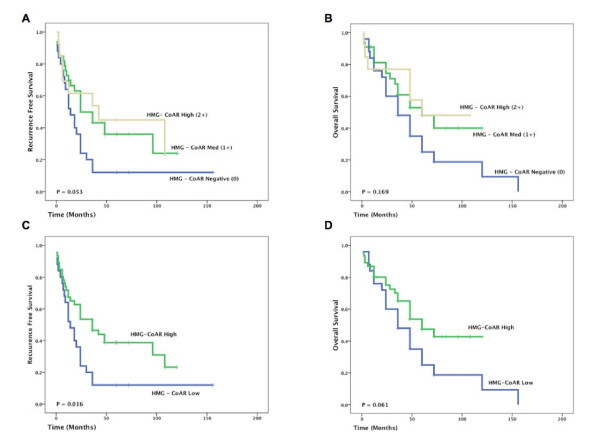
**HMG-CoAR is Associated with Prolonged RFS in EOC**. Kaplan Meier analysis of manually assessed HMG-CoAR cytoplasmic intensity revealed a trend towards an improved RFS (A) and OS (B). Dichotomization of data as positive versus negative revealed that HMG-CoAR was associated with an improved RFS (C) but not an improved OS (D).

Having demonstrated that HMG-CoAR was differentially expressed in EOC, the relationship between HMG-CoAR expression and prognosis was evaluated. As tumours were arrayed in quadruplicate, median expression values were used for survival analysis. Kaplan Meier analysis demonstrated that HMG-CoAR was associated with a non-significant stepwise improvement in both RFS (Fig. 2A) and OS (Fig. 2B). Patients with moderate (2+) HMG-CoAR expression had a median RFS of 42 months compared to 14 months for patients with HMG-CoAR-negative tumours.

Based on these findings a dichotomized variable comparing absent versus any staining was defined. This revealed that HMG-CoAR expression was associated with a prolonged RFS (p = 0.016) and a trend towards a prolonged OS (p = 0.061). Cox univariate analysis (Table 1) confirmed that HMG-CoAR expression was associated with an improved RFS (HR = 0.52, 95% CI 0.30 - 0.91, p = 0.022) and multivariate regression analysis of RFS revealed that after adjusting for stage and grade, HMG-CoAR was still a significant predictor of improved RFS (HR = 0.54, 95% CI 0.30 - 0.96, p = 0.036) (Table 1).

**Table 1:** Cox regression analysis of RFS based on manual and automated assessment of HMG-CoAR expression.

	**Manual Analysis**		**Autoscore Continuous**		**Autoscore Dichotomised**	
			
	**HR (95%CI)**	***P value***	**HR (95%CI)**	***P value***	**HR (95%CI)**	***P value***
			
	***Univariate***		***Univariate***		***Univariate***	
**HMG-CoAR** (*high versus low*)	0.52 (0.30-0.91)	0.022	0.98 (0.97 -- 0.99)	0.039	0.47 (0.25 -- 0.87)	0.017
**Stage** (*continuous*)	2.17 (1.16-4.03)	0.015	2.17 (1.16 - 4.03)	0.015	2.17 (1.16 - 4.03)	0.015
**Grade** (*Low versus moderate and high*)	1.32 (0.62-2.81)	0.471	1.32 (0.62 - 2.81)	0.471	1.32 (0.62 - 2.81)	0.471
**Resdiual Disease** (*no macrscopic disease v's macroscopic disease*)	0.79 (0.35-1.81)	0.58	0.79 (0.35 - 1.81)	0.58	0.79 (0.35 - 1.81)	0.58
						
	Multivariate*		Multivariate*		Multivariate*	
**HMG-CoAR** (*high versus low*)	0.52 (0.30-0.96)	0.036	0.99 (0.97 -- 0.99)	0.04	0.49 (0.25 -- 0.99)	0.03
**Stage** (continuous)	1.31 (0.61-2.80)	0.485	1.33 (0.64-2.77)	0.447	1.40 (0.66 - 2.95)	0.373
**Grade** (*Low versus moderate and high*)	2.03 (0.66-6.27)	0.216	1.15 (0.43-3.07)	0.777	1.28 (0.49 - 3.29)	0.61
**Resdiual Disease** (*no macrscopic disease v's macroscopic disease*)	0.40 (0.12 1.29)	0.123	0.87 (0.53 - 1.45)	0.597	0.88 (0.53 - 1.50)	0.621

### Automated Analysis Confirms HMG-CoAR as a Good Prognostic Marker

**Figure 3:**
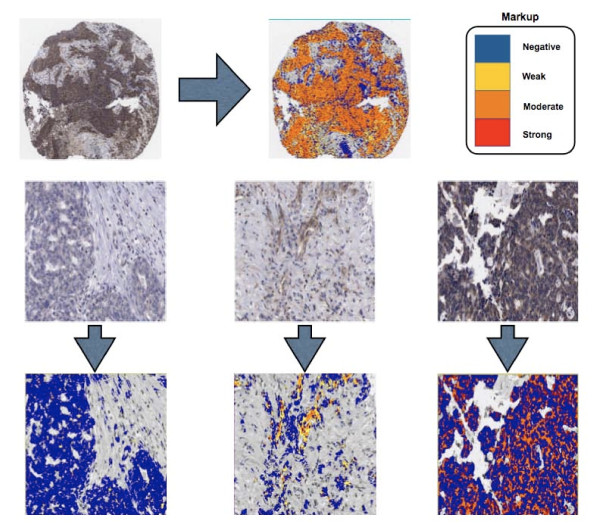
**Automated Analysis of HMG-CoAR Protein Expression**. Using Genie pattern recognitiion software, tumour and stroma were identified and tumour specific HMG-CoAR was quantified using a colour deconvolution algorithm. The images shown are IHC and mark-up images, markups show different levels of HMG-CoAR as described by the colour coded legend.

Our group has previously demonstrated that automated analysis of IHC can identify new prognostic subgroups [15-17], and automated analysis was used in this study to develop a quantitative scoring model for HMG-CoAR expression in EOC. The approach adopted in this study differed from previous experiments as pattern recognition software (Genie, Aperio) was initially used to identify tumour from stroma and then tumour-specific HMG-CoAR expression was quantified using a postive pixel count algorithm. The output of the algorithm was staining intensity and percentage positive tumour cells. The approach is illustrated in Fig. 3.

**Figure 4:**
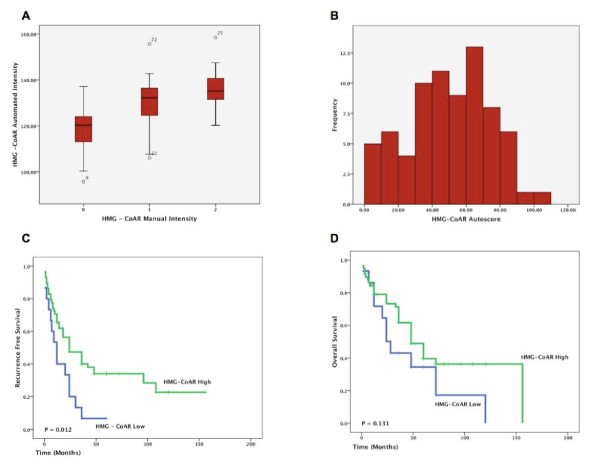
**HMG-CoAR Autoscore is Associated with an Improved RFS**. There was an excellent correlation between automated and manual cytoplasmic intensity (A). A HMG-CoAR autoscore was calculated by combining cytoplasmic intensity and the percentage of positive tumour cells. The distribution of the HMG-CoAR autoscore is illustrated in the histogram (B). Using a threshold of the 25th percentile, an increased HMG-CoAR autoscore was associated with a prolonged RFS (C) but not OS (D).

A strong correlation was evident between manual and automated analysis of staining intensity (r = 0.61, p < 0.001) (Fig. 4A). Automated intensity values of duplicate cores from individual tumour blocks showed an excellent correlation (Spearman's Rho 0.763, p < 0.001) suggesting a homogenous pattern of expression of HMG-CoAR in EOC and thus making it suitable for TMA-based analysis.

Using automated analysis an HMG-CoAR autoscore combining intensity and percentage positive tumour cells was developed. As specimens were arrayed in quadruplicate a median HMG-CoAR autoscore was calculated for each tumour. The distribution of the HMG-CoAR autoscore is illustrated in Fig. 4B. Cox univariate analysis of the HMG-CoAR autoscore as a continuous value revealed that it was associated with an improved RFS (HR = 0.98, 95% CI 0.97 - 0.99, p = 0.039) (Table 1). No relationship was seen between HMG-CoAR autoscore and OS (HR = 0.99, 95% CI 0.98 - 1.01, p = 0.41). Cox multivariate analysis of HMG-CoAR autoscore as a continuous variable confirmed increased expression of HMG-CoAR protein was associated with an improved RFS after controlling for stage and grade (HR = 0.98, 95% CI 0.97 - 0.99, p = 0.040) (Table 1).

HMG-CoAR autosore was then dichotomised using the 25^th ^percentile (corresponding to an autoscore value of 35) as a threshold. Kaplan Meier analysis of the HMG-CoAR as a dichotomised value demonstrated that increased levels of HMG-CoAR protein expression were associated with an improved RFS (p = 0.012) (Fig. 4C). A high HMG-CoAR autoscore was associated with a non-significant trend towards an improved OS (p = 0.131) (Fig. 4D). Cox univariate analysis of dichotomised HMG-CoAR autoscore confirmed the association between HMG-CoAR protein expression and a prolonged RFS (HR = 0.47, 95% CI 0.25 - 0.87, p = 0.017). Cox multivariate analysis controlling for grade, stage and residual disease revealed that increased levels of HMG-CoAR protein expression, as demonstrated by a high HMG-CoAR autoscore, was an independent predictor of a RFS in EC (HR = 0.49, 95% CI 0.25 - 0.993, p = 0.03) (Table 1). No relationship was evident between HMG-CoAR expression and age, grade, stage, histological subtype, estrogen receptor or Ki-67 status (Table 2).

**Table 2:** Patient and tumour characteristics stratified according to HMG-CoAR status

	HMG-CoAR Low (n = 16)	HMG-CoAR High (n = 56)	P Value
**Age**			
Mean (SEM)	52.2 (1.96)	53.7 (1.70)	0.56
			
**Histology**			
Serous	12 (80)	36 (64)	0.358
Non Serous	4 (20	20 (36)	
			
**Grade**			
Well Differentiated	1 (6)	11 (20)	0.858
Moderately Differentiated	9 (56)	18 (32)	
Poorly Differentiated	6 (38)	27 (38)	
			
**Stage**			
1	0	0	
2	4 (25)	16 (29)	
3	12 (75)	39 (70)	
4	0	1 (1)	
			
**Estrogen Receptor**			
0-10%	6 (38)	10 (18)	0.217
11-100%	10 (62)	46 (82)	
			
**Ki-67**			
0-10%	4 (25)	7 (13)	0.411
11-100%	12 (75)	49 (87)	

## Discussion

This is, to our knowledge, the first study to describe tumour-specific HMG-CoAR expression in EOC. Cytoplasmic expression of HMG-CoAR was evident in varying intensities in 65% of the tumours. Although HMG-CoAR was not associated with disease stage, grade, estrogen receptor or Ki-67 expression, it was associated with a prolonged RFS. Manual and automated quantification of HMG-CoAR expression were both associated with a prolonged RFS and Cox multivariate proportional hazards analysis confirmed that this was independent of stage and grade. These findings support previous results from our group describing the association between tumour-specific HMG-CoAR expression in breast cancer and a less aggressive tumour phenotype [13,14].

As HMG-CoAR is the rate-limiting enzyme of the mevalonate pathway, these data add further evidence of this pathway's importance in tumour development and progression. While HMG-CoAR inhibitors, also known as statins, have demonstrated excellent efficacy in the treatment of hypercholesterolemia and cardiovascular disease, their role in oncology remains relatively unproven. Despite an ever-growing body of literature describing the anti-neoplastic properties of statins, epidemiologic data regarding their preventive effect against cancer in general, and EOC in particular, remain inconclusive [9,10,18-22]. A recent pre-operative window trial of ductal carcinoma in situ and stage one breast cancer was the first to demonstrate that statins can inhibit proliferation and increase apoptosis *in vivo *[23]. This raises the possibility that the combination of statins and well-established chemotherapeutic agents may be an option in the neo-adjuvant setting in other tumour types also.

HMG-CoAR activity in tumour cells is elevated and dysregulated. HMG-CoAR activity in leukemia cells [24,25] and lung carcinoma cells [26] are 3-8-fold and 2-fold higher, respectively, than in normal cells. Furthermore, statin induced mevalonate depletion has been shown to result in an adaptive induction of HMG-CoAR expression in chinese hamster ovary cells [27] and MCF-7 breast cancer cells [28]. Treatment of MCF-7 cells with mevastatin resulted in a 10- to 15-fold induction of HMG-CoAR activity in association with a 2.5- to 3.5-fold induction of HMG-CoA reductase mRNA expression [28], suggesting that treatment with statins may increase tumour specific HMG-CoAR expression *in vivo*, however this remains to be fully elucidated. It seems counterintuitive that statins cause an increase in tumour-specifc HMG-CoAR expression however this is felt to be secondary to a loss of sterol mediated inhibition of HMG-CoAR transcription in tumour cells [2]. The statin induced increase in HMG-CoAR results in an increase non-sterol isoprenoid side products, with their associated tumour-suppressive properties, which may explain the efficacy of statin in treating tumour cells *in vitro *[2]

Kato *et al *recently demonstrated that lypophillic statins induce apoptosis in ovarian cancer cells, and also postulated that HMG-CoAR expression predicted response to statin treatment [29]. In vitro data demonstrate that statins induce apoptosis and inhibit tumour formation in soft agar in ovarian cancer cells via activation of the JNK pathway and pro-apoptotic proteins such as Bim [30]. Additionally statin induced suppression of RhoA has been shown to inhibit peritoneal dissemination of ovarian cancer cells *in vivo *[31]. Likewise high-dose lovastatin has been shown to inhibit tumour proliferation in a xenograft model of anaplastic thyroid cancer [32].

It has been postulated that the anti-neoplastic effects of statins could be attributed to their ability to increase HMG-CoAR activity in tumour cells, thus leading to the production of non-sterol bi-products of the mevalonate pathway [2]. Increased HMG-CoAR activity increases the synthesis of farnesyl diphosphate and geranylgeranyl diphosphate. These substrates provide the isoprenoid moieties for the post-translational modification of the cysteine residue of the conserved carboxyl terminus sequence of diverse proteins - known as prenylation [2]. Prenylation has been shown to have a number of tumour suppressive actions including the induction of apoptosis [33], the initiation of G1 arrest [33] and the suppression of small G-protein receptors and intracellular growth pathways [34]. HMG-CoAR expression could be a surrogate marker of protein prenylation, thus explaining our findings that increased levels of HMG-CoaR are associated with an improved prognosis in both breast and EOC.

## Conclusion

In summary, this is the first description of tumour-specific HMG-CoAR expression in EOC. Given that all of the patients in this study received adjuvant platinum-based chemotherapy, these data suggest that the addition of statins to traditional chemotherapeutic regimens may be an efficacious and well-tolerated strategy in EOC. Although data were not available on statin use in this cohort, a growing body of experimental evidence exists describing a synergism between cisplatinum and statins in vitro [35-37]. Recent *in vivo *data confirmed that statins have an anti-neoplastic effect in breast cancer [23] and it is anticipated that ongoing prospective trials will shed more light on this issue [38]. It should also be noted that while further studies are required to investigate the value of HMG-CoAR expression as a predictive marker of response to statin treatment, our results provide evidence to justify prospective randomized controlled trials examining the addition of statins to standard adjuvant chemotherapeutic regimens for EOC.

## Competing interests

The authors declare that they have no competing interests.

## Authors' contributions

DJB designed the study, performed image analysis, statistical analysis and drafted the manuscript, JB performed manual analysis of IHC and statistical analysis, ER performed image analysis, MF developed the patient database, FP designed the study and helped draft the manuscript, MU designed the study and helped draft the manuscript, WG provided image analysis platforms and designed the study, DO'C designed the study and drafted the manuscript, CO'H provided TMAs and designed the study, KJ performed IHC, manual analysis and drafted the manuscript. All authors read and approved the final manuscript.

## Authors Information

DJB, JB, MF and CO'H are practising obstetricians and gynaecologists, DJB, ER, DO'C, WG and MU are research scientists with a special interest in automated quantification of immunohistochemistry, FP and KJ are practising pathologists.

## Pre-publication history

The pre-publication history for this paper can be accessed here:

http://www.biomedcentral.com/1471-2407/10/125/prepub
